# Long-Term Effects of Serial Anodal tDCS on Motion Perception in Subjects with Occipital Stroke Measured in the Unaffected Visual Hemifield

**DOI:** 10.3389/fnhum.2013.00314

**Published:** 2013-06-24

**Authors:** M. C. Olma, R. A. Dargie, J. R. Behrens, A. Kraft, K. Irlbacher, M. Fahle, S. A. Brandt

**Affiliations:** ^1^Department of Neurology, Charité University Hospital, Berlin, Germany; ^2^The College of Medicine and Veterinary Medicine, The University of Edinburgh, Edinburgh, UK; ^3^Department of Human Neurobiology, University of Bremen, Bremen, Germany

**Keywords:** transcranial direct current stimulation, visual system, motor system, translational research, motion perception, neuroplasticity, learning

## Abstract

Transcranial direct current stimulation (tDCS) is a novel neuromodulatory tool that has seen early transition to clinical trials, although the high variability of these findings necessitates further studies in clinically relevant populations. The majority of evidence into effects of repeated tDCS is based on research in the human motor system, but it is unclear whether the long-term effects of serial tDCS are motor-specific or transferable to other brain areas. This study aimed to examine whether serial anodal tDCS over the visual cortex can exogenously induce long-term neuroplastic changes in the visual cortex. However, when the visual cortex is affected by a cortical lesion, up-regulated endogenous neuroplastic adaptation processes may alter the susceptibility to tDCS. To this end, motion perception was investigated in the unaffected hemifield of subjects with unilateral visual cortex lesions. Twelve subjects with occipital ischemic lesions participated in a within-subject, sham-controlled, double-blind study. MRI-registered sham or anodal tDCS (1.5 mA, 20 min) was applied on five consecutive days over the visual cortex. Motion perception was tested before and after stimulation sessions and at 14- and 28-day follow-up. After a 16-day interval an identical study block with the other stimulation condition (anodal or sham tDCS) followed. Serial anodal tDCS over the visual cortex resulted in an improvement in motion perception, a function attributed to MT/V5. This effect was still measurable at 14- and 28-day follow-up measurements. Thus, this may represent evidence for long-term tDCS-induced plasticity and has implications for the design of studies examining the time course of tDCS effects in both the visual and motor systems.

## Introduction

Arguably the greatest future challenge facing transcranial direct current stimulation (tDCS), a promising tool for non-invasive neuromodulation, will be its effective translation to clinical use. However, evidence suggests that changes in connectivity and neuroplasticity in the aging or lesioned brain may have different prerequisites and mechanisms than similar processes in younger healthy systems (DeCarli et al., 2012; Grady, 2012). This raises the question of how directly tDCS-induced synaptic plasticity documented by numerous studies in animals and healthy young subjects will manifest in these subject groups, and to what extent persistent, long-term (weeks–months) and clinically relevant effects can be achieved.

The effects of tDCS on the human motor-cortex are well-established. Single-session stimulation with weak anodal or cathodal transcranial direct current (current densities of<0.1 mA/cm^2^) respectively lowers and raises the threshold to motor-evoked potential (MEP) induction by transcranial magnetic stimulation (TMS), with effects outlasting the stimulation period by up to 90 min (Priori et al., 1998; Nitsche and Paulus, 2000, 2001; Rosenkranz et al., 2000; Quartarone et al., 2004; Furubayashi et al., 2008).

Pharmacological studies deepened our understanding of these effects and their relation to other forms of neuroplasticity (Gartside, 1968; Bailey et al., 2000; Liebetanz et al., 2002; Nitsche et al., 2003a, 2004). These collective findings strongly suggest that LTP-like and LTD-like processes underlie the observed neuroplastic effects of tDCS (Nitsche et al., 2008; Stagg and Nitsche, 2011).

Early animal work into direct current on exposed cortical surfaces reported similar findings in the motor and visual systems (Bindman et al., 1962, 1964; Creutzfeldt et al., 1962; Landau et al., 1964). TDCS studies in humans could demonstrate that excitability of the visual cortex, measured, e.g., by visual-evoked potentials (VEPs), can be elevated by occipital anodal tDCS and lowered by cathodal tDCS especially when low signal-to-noise stimuli are used (Antal et al., 2004a; Accornero et al., 2007; Lang et al., 2007). Studies utilizing low signal-to-noise perceptual threshold tasks detected significant anodal tDCS effects on visual contrast sensitivity (Kraft et al., 2010a; Olma et al., 2011), while a similar study identified no stimulation effects of anodal tDCS when supra-threshold stimuli were used (Antal et al., 2001). This suggests that threshold measures are well-suited to further assess tDCS in this system. Although the immediate effects of tDCS in the healthy visual system are of shorter duration than in the motor system – likely to be related to the interaction of current fields with gross cranial anatomy, cortical microarchitecture, and underlying system activity (Nitsche et al., 2008) – long-term effects of repeated tDCS may be translatable from the motor to the visual system (Antal and Paulus, 2008; Antal et al., 2011).

To this end, we investigated the immediate and long-term effects of anodal serial tDCS on an established motion detection threshold paradigm (Antal et al., 2004c; Kraft et al., 2010b). However, endogenous neuroplastic adaptation processes following a cortical lesion in the visual cortex may be up-regulated and alter the susceptibility to tDCS. Therefore, we investigated subjects suffering chronic visual-system ischemic strokes to model the presence of lesions in target stimulation populations. MRI-navigated anodal or sham tDCS was administered on five consecutive days over the ipsilesional visual cortex. Motion perception thresholds of the unaffected visual field (corresponding the contralesional hemisphere, to provide data comparable with previous studies in healthy subjects) were measured directly before and after each stimulation session, then again at 2- and 4-week follow-up after the fifth stimulation day, within a double-blinded, within-subject cross-over study.

## Materials and Methods

### Study participants

Twelve subjects [mean age 53.5 ± 14.9 (SD) years; six female; all right-handed] participated in the study. All had a history of ischemic stroke and chronic homonymous visual field defects (hVFD; four homonymous hemianopias, seven homonymous quadrantanopias, one homonymous paracentral scotoma). In all subjects, no transient or persistent ischemic attacks had been reported or diagnosed in the 6 months preceding inclusion in the study. Subjects were thus in comparable chronic post-stroke phases, when nil or minimal spontaneous changes in visual perception are expected (Zhang et al., 2006). Mean interval between stroke occurrence and inclusion in the study was 18.4 ± 8.2 (SD) months. Occipital lesions were relatively small, well-defined unilateral posterior infarcts and – importantly – there remained intact cortex at the occipital pole (stimulation site): mean lesion volume was 8.4 (±7.0 SD) cm^3^ and mean minimum distance from the internal surface of the occipital pole cranium was 14.6 (±6.9 SD) mm. Subjects had no history of hemorrhagic or traumatic brain injury, or progressive neurological disease, no ophthalmological or pre-chiasmal disorders, no psychiatric disease, cognitive impairment [as measured by Montreal Cognitive Assessment (MoCA, Nasreddine et al., 2005)], or history of substance abuse. Unilateral hemispatial neglect was excluded by assessment with clock-drawing, image-replication, line-bisection, and star-cancelation tasks. Each subject scored ≥3 out of 5 in the visuo-spatial section and ≥4 out of 6 in the attention section of the MoCA. Full function of the dominant (right) hand was present in all subjects. No subjects had metal cranial implants, cardiac pacemakers, or other implanted devices. Prior to inclusion in the study, standard automated perimetry confirmed congruent, homonymous visual field defects in all subjects. Standard 10–20 electroencephalogram of each subject was free of epileptiform potentials at rest and on graded photic and hyperventilation provocation. All subjects had normal or corrected-to-normal visual acuity and had not received structured visual rehabilitation. The study conformed to the Declaration of Helsinki and was approved by the local ethics committee. Each subject provided written, informed consent and received financial compensation to cover travel costs to appointments.

### Experimental procedure

Experiments were performed before 12 noon to control for daily fluctuations in concentration and visual discrimination (Bonnefond et al., 2003; Schmidt et al., 2007). All experiments were performed in the same darkened room. At the start of each experimental test day, subjects sat quietly for 5 min to allow dark adaptation. The order of experimental procedure was as follows: computerized campimetric tests [i.e., performed on a flat-screen, not a perimetric bowl (Kraft et al., 2010b)] of color and motion detection (3 min each), automated threshold perimetry (6 min), 20 min intervention (anodal or sham tDCS), perimetric tests (6 min), and computerized campimetric tests of color and motion perception (3 min each). The order of color and motion campimetric tests was randomized daily and balanced over the subject group. Results from the color campimetric test and contrast perimetry shall be reported elsewhere.

### Stimuli, task, and procedure

Details of the alternative forced-choice motion detection paradigm used have been previously published (Kraft et al., 2010b) and are thus only briefly outlined here. The test was performed on a 21″ diameter, 1600 × 1200 pixel, 75 Hz Iiyama monitor connected to a personal computer. Subjects’ heads were stabilized on an adjustable chin rest 60 cm from the monitor, the height of which was adjusted so that subject eye level was at the height of the monitor’s central point. Subjects were required to maintain fixation of a small (12.0 arcmin) central dot throughout the full duration of both campimetric tests.

**Figure 1:**
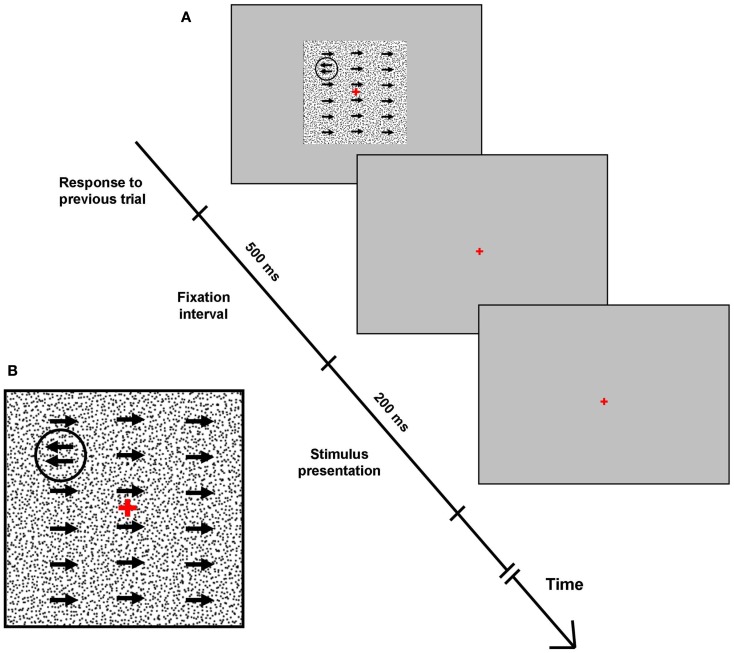
**Motion detection task**. **(A)** Full-screen screenshots of the campimetric motion detection perceptual task. Each response was followed by a 500 ms fixation screen, before the test screen with background and quadrant-specific stimulus were presented for 200 ms (see text for stimulus and background details). Following this, subjects had unlimited time to select one quadrant via a four-button keypad. Arrows signify coherent movement of 5 acrmin diameter monochromatic dots. **(B)** Inset: close-up of test screen.

The stimuli for the motion detection task comprised a gray background (54 cd/m^2^), against which 20,000 black dots of 5.0 arcmin diameter moved to the right at a velocity of 3°/s. A monochromatic circle of 210.0 arcmin diameter, composed of dots of the same two intensities but moving to the left with variable velocity, was presented for 200 ms in one (random) visual field quadrant at 5° eccentricity from fixation (Figure 1).

Subject’s task was to indicate the location of the circle via a manual button press using a four-button keypad (four-alternative-forced-choice task). After stimulus presentation, subjects had unlimited time to select the quadrant they deemed the stimulus was presented in. Incorrect responses triggered a short tone as error feedback, and each response elicited the next stimulus at an interval of 500 ms. Stimulus dots were initially set to move to the left with a velocity of 3°/s, producing an easily detectable circular stimulus (Figure 1). As the test progressed and subjects provided correct answers in each quadrant, the difference between the motion direction of background dots and stimulus dots (i.e., stimulus/background contrast) decreased, and stimuli became more difficult to detect.

This allowed a quadrant-specific motion detection threshold to be attained, the contrast level (in degrees of stimulus movement direction) at which 62.5% responses were correct, based on the adaptive staircase procedure Quick Estimation by Sequential Testing (QUEST) (Watson and Pelli, 1983; Kraft et al., 2010b). All tests comprised 120 trials (30 per visual field quadrant) and lasted approximately 3 min. The test was performed binocularly. No formal fixation control was applied, but the short stimulus presentation duration (200 ms) and investigator eye-movement observation via an adjustable mirror provided basic controls. Subjects were reassured that it was normal and necessary not to perceive all stimuli and encouraged to give intuitive responses, using all quadrants, when unsure. This aimed to reduce bias toward any particular quadrant. Nevertheless, the probability of guessing the right target quadrant was theoretically 25%, with a measured standard deviation of 6.8% over the entire group.

However, some subjects with stroke-related visual field defects in specific quadrants could not or could only poorly perceive the motion stimuli there. Grouping “perceivers,” “poor-perceivers,” and “not-perceivers” into one group would cause a high heterogeneity confounding the interpretation of the tDCS effect in these ipsilesional quadrants. Therefore, data from the ipsilesional quadrants were excluded from the analysis in this study.

Threshold values for quadrants that subjects selected too seldomly for a threshold to be accurately calculated, or in which a threshold value greater than the highest possible value (180°) was calculated, were replaced with the value corresponding to the first stimulus presented, 180°(Kraft et al., 2010b). Perceptual thresholds were conducted using *post hoc* fitting procedures (Probit-analysis). Age-corrected threshold values were obtained for all datapoints by subtracting the subject’s actual motion thresholds from the motion thresholds that would be expected for a given age range (Kraft et al., 2010b). A negative result of this difference signifies that the motion sensitivity was below the age-norm, i.e., performing poorer than the age-norm. A positive difference of this age correction indicates a performance above the age-norm. This deviation from the age-norm of motion sensitivity will be referred to as “Δ motion sensitivity” and was entered as the depending variable into the statistical analyses.

### Transcranial direct current stimulation: Electrode position

**Figure 2:**
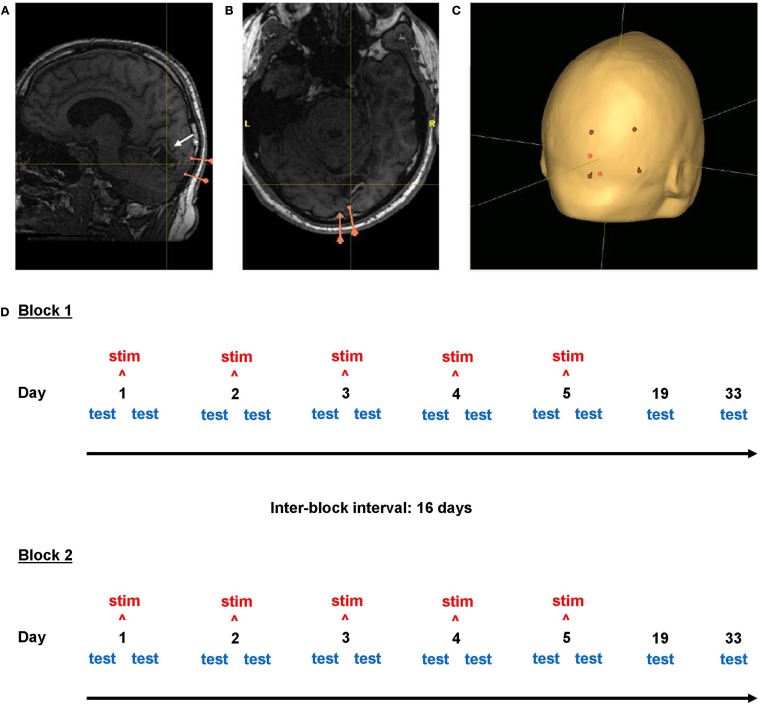
**Neuronavigation and study design**. Screenshots from the neuronavigational software Nexstim Eximia. **(A,B)** Occipital lobe lesion in one subject (center of cross-hairs). Pink markers represent the two meningeal landmarks used for electrode positioning. **(C)** Scalp localization (red) of the corners of the 5 cm × 7 cm stimulation electrode above the ipsilesional calcarine sulcus (white arrow). Cross-hair location is constant in **(A–C)**. The study design is illustrated in **(D)**.

In order to control for individual variation in skull and posterior lobe anatomy, to maximize stimulation of the primary visual cortex and to provide a clinically relevant stimulation setting, the scalp directly superficial to the ipsilesional calcarine sulcus was selected to be the site of the stimulation electrode (Figure 2). Positioning was achieved using the navigation-system Nexstim Eximia Navigated Brain Stimulation System (Nexstim, Helsinki, Finland; see Schmidt et al., 2009), allowing co-registration of the subjects’ heads in 3-D space with the corresponding anatomical MRI data using an infra-red camera (Polaris Spectra, Northern Digital Inc., ON, Canada) and 38 mm infra-red spectacles (Oculus, Wetzlar, Germany). A 1.5 T Magnetom Vision MRI scanner (Siemens, Erlangen, Germany) was used to acquire T1-weighted magnetization-prepared rapid gradient-echo sampling (MP RAGE) sequences for each subject (Brant-Zawadzki and Gillan, 1992; Howarth et al., 2006). The lower horizontal border of the 5 cm× 5 cm anodal electrode was defined by a scalp point superficial to the tentorium cerebelli; the medial vertical border of the anodal electrode position was defined by a scalp point superficial to the brain location 1 cm lateral to the interhemispheric falx cerebri (Figure 2). The midpoint of the 7 cm × 5 cm reference cathodal electrode lay over the 20–10 electrode position Cz. Electrode positions were delineated with permanent marker pen on the subject’s head and renewed at the start of each intervention day to maintain correct electrode position without daily MRI-navigation.

### Stimulation

Anodal and sham tDCS was administered using an Eldith Direct Current Stimulator Plus (Neuroconn, Ilmenau, Germany), via two non-metallic conductive rubber electrodes ensheathed in synthetic sponges, which were soaked in 0.9% Na^+^Cl^−^ to reduce electrical resistance and subject discomfort, a key requirement of double-blind sham-controlled tDCS studies (Gandiga et al., 2006; Dundas et al., 2007). This solution was also carefully applied to subjects’ scalps at electrode positions, and large hair strands dispersed. Stimulation was to cease automatically if circuit resistance exceeded 40 kΩ; outwith the initial and final 15 s of stimulation (current “ramping” phase, see below) resistance did not exceed 8 kΩ for any subject. Electrodes were secured in place with a non-rubber headband and self-adhesive medical bandage. The stimulating electrode had a surface area of 25 cm^2^, the reference of 35 cm^2^.

Anodal and sham tDCS were administered for 20 min during stimulation sessions. Anodal tDCS was applied at a current of 1.5 mA, giving a current density of 0.06 mA/cm^2^, within published safety recommendations (Iyer et al., 2005; Poreisz et al., 2007; Williams et al., 2009; Kessler et al., 2012). At this current density, an initial skin tingling under the anodal electrode may be expected on initiation of tDCS (Paulus, 2003). For this reason, gradual current “ramping” was employed in the first 15 s of both anodal and sham tDCS (Hummel et al., 2008), after which 1.5 mA stimulation followed for 20 min in the anodal condition and no stimulation followed for 20 min in the sham condition. Current was ramped down for the final 15 s at the end of each 20-min sham and anodal stimulation session. Each 5-day intervention week was allocated a five-digit code, corresponding either to anodal or sham stimulation, by a supervising researcher who did not perform any experimental work. Thus the direct investigators were experimentally blinded to the stimulation condition. Subjects were informed of possible mild side-effects such as skin irritation, tingling under the electrodes, and mild headache, and encouraged to report any unpleasantness or discomfort.

### Study design

The study followed a within-subject, repeated-measures, cross-over design, comprising two blocks, each with a 5-day stimulation week with daily measurements, then two follow-up measurements at 2 and 4 weeks (Figure 2: Study Design). Subjects received either anodal or sham tDCS on five consecutive days, with visual testing before and after intervention. Measurements are described below with reference to study day and as “pre” or “post” (before or after intervention, only relevant for days 1–5). Subjects returned 2 weeks (day 19) and 4 weeks (day 33) later for follow-up measurements. This gave a total of 12 measurements per block (Figure 2). Long-term effects of anodal tDCS lasting up to 33 days were considered possible. To minimize the possibility of long-term effects of anodal tDCS (if administered in the first block) influencing the sham condition, a 16-day interval was observed between the last measurement of block one and the first stimulation day of block two; thus the total time interval between 5-day intervention weeks was 6 weeks, and total study duration per subject was 12 weeks (Figure 2). The second block followed an identical schedule as the first. Each subject received both anodal and sham intervention weeks and the two stimulation conditions were counterbalanced between blocks: six subjects received anodal tDCS in the first block, while six received it in the second block.

### Data analysis

All analyses were performed with IBM SPSS Statistics 19 for Windows. Age-corrected motion threshold values, generated by the subtraction of the subjects’ motion threshold from decade-sensitive normal reference values data (= Δ motion sensitivity, see above), were used for analysis. Due to a small number of missing values (see Results), using a traditional statistical analysis such as ANOVA with repeated measures would have led to a substantial decrease in statistical power. As an alternative statistical analysis of Δ motion sensitivity that is unaffected by few missing data, a linear mixed model (LMM) with subjects as random factor was used (Shek and Ma, 2011). The following variables were used to setup our statistical models. The covariate “stimulation” was coded 0 for sham and 1 for anodal stimulation. The covariate “time” was coded 1, 2, 3, 4, 5, 19, and 33 for the respective days. The covariate “sequence” was coded as 0 for the first block and 1 for the second block. The covariate “intervention” was coded as 0 for the measurement before the stimulation and 1 the measurement afterward. The covariate “quadrant” was coded as 0 for the lower quadrant and 1 for the upper quadrant. A *t*-test for independent groups was used to analyze potential baseline differences between the groups that started either with anodal tDCS or sham tDCS. In addition, a bivariate correlation analysis was performed to test whether the stimulation effect correlated with the lesion volume or the distance from the posterior lesion margin to the internal surface of the occipital pole cranium (Pearson correlation, two-tailed, *p* < 0.05). The stimulation effect was calculated across the stimulation week as the difference between baseline measurement (day 1, before stimulation) and Δ motion sensitivity at the last day of the stimulation week (day 5, after stimulation). Accordingly, the long-term stimulation effect was calculated as the difference between baseline measurement (day 1, before stimulation) and Δ motion sensitivity at the last day of the follow-up measurement (day 33).

## Results

Blinding to stimulation condition was successful: nine subjects reported not knowing whether they received anodal or sham tDCS in each block; of the remaining three, only one guessed correctly, with “moderate certainty,” not because of feeling the stimulation, but due to a subjective improvement in peripheral visual field sensitivity within the anodal block.

All procedures were well-tolerated by subjects and no serious known side-effects of tDCS were reported at any point. One subject with pre-existing scalp xerosis reported irritation during intervention sessions in both anodal and sham blocks, most likely due to 0.9% Na^+^Cl^−^ solution. A second subject with a previous history of intermittent piercing chest pain experienced a recurrence of chest pain on the third day of the sham block, not thought to be connected to the present study. No subjects reported deterioration in vision (sensitivity, acuity, resolution, color, movement, or general vision).

The parameter Δ motion sensitivity obtained at all measurements by all 12 subjects was approximately normally distributed. From the 576 possible total data points (12 subjects × 12 time points × 2 stimulations × 2 unaffected quadrants), 10 measurements (1.74%) were lost because a subject could not attend on the scheduled day. A *t*-test for independent groups did not reveal a significant difference in motor sensitivity between the anodal and sham condition at baseline of the first block (*p* = 0.266).

The LMM specified to model the effects of serial tDCS on Δ motion sensitivity within the stimulation week included the repeated covariates “stimulation” (anodal, sham), “intervention” (pre, post), “time” (day 1, day 2, day 3, day 4, day 5), and “quadrant” (superior, inferior) (Table 1). With regard to the within-subject design, the learning state between block one and block two differed (being less experienced in the first block and more experienced in the second block). Since this was expected to affect the performance of the motion detection task, the covariate “sequence” (block 1, block 2) was introduced into the LMM.

**Table 1:** **Linear mixed model statistics from the stimulation week of the study**.

Parameter	Estimate	Std. error	*p*-Value	
	
Intercept	0.90	2.62	0.74	
Stimulation	1.11	0.57	0.05	
Time	0.99	0.20	<0.01	
Intervention	−0.71	0.57	0.22	
Quadrant	2.76	0.57	<0.01	
Sequence	5.31	0.57	<0.01	
Variance components		SD		
Subjects		6.20		

**Figure 3:**
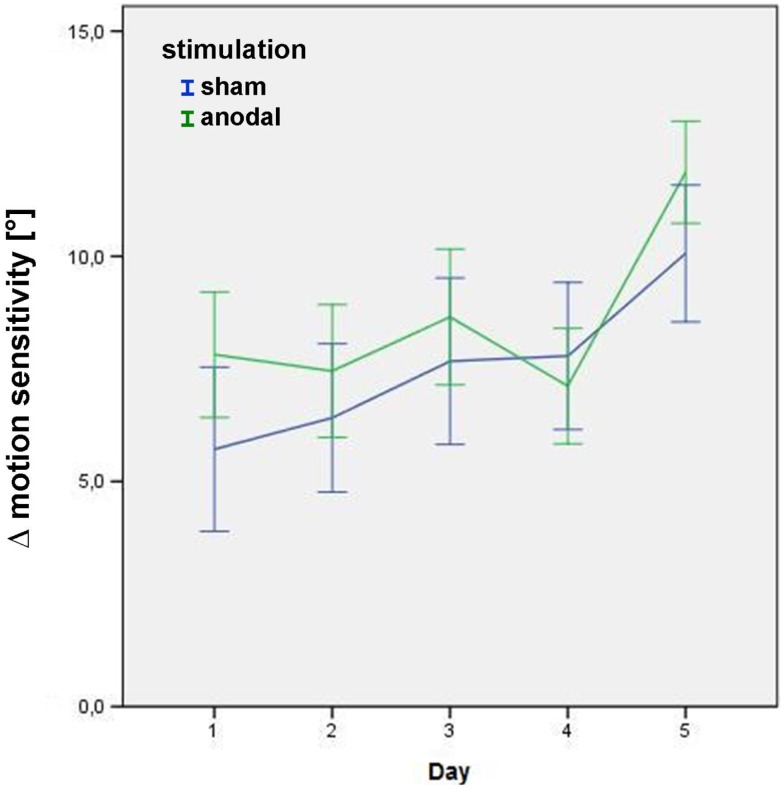
**Δ motion sensitivity during the stimulation week**. Data from the post-stimulation motion detection tests on days 1–5 in the study block, showing improvement in age-matched motion detection [subtraction of the subjects’ motion threshold from decade-sensitive normal reference values data = Δ motion sensitivity (in°)] in both the sham (blue) and anodal (green) conditions. Error bars display one standard error of the mean. There was no significant baseline difference between groups (*p* = 0.266). See text for details of the significant main effects stimulation, day, and quadrant within the linear mixed model.

Significant main effects were found for “stimulation” (*p* = 0.05), and “time” (*p* < 0.01), i.e., Δ motion sensitivity increased across the stimulation week in both stimulation conditions (= learning effect), with an additional increase of Δ motion sensitivity for the anodal stimulation condition (Figure 3), even when a sequence effect is present with generally higher levels of Δ motion sensitivity in the second block (*p* < 0.01). Interestingly, the immediate intervention effect did not reach significance (*p* = 0.22), indicating that the improvement of Δ motion sensitivity was partially mediated through an overnight effect. There was a significant main effect for “quadrant” (*p* < 0.01), describing how greater changes in Δ motion sensitivity were attained in the upper visual field quadrant.

In order to analyze the potential long-term effects of serial tDCS, the parameter Δ motion sensitivity measured post-stimulation was entered in another LMM (Table 2), including the covariates “time” (day 1, day 2, day 3, day 4, day 5, day 19, day 33), “stimulation” (anodal, sham), and “quadrant” (superior, inferior). Only one test per day of stimulation block (post-stimulation) was included, because the follow-up period comprises only post-stimulation measurements. In order to adjust for a sequence effect between blocks, the covariate “sequence” was included. Since the learning curve may decline throughout the experiment the interaction “sequence × time” was introduced into the LMM.

**Table 2:** **Linear mixed model statistics from stimulation week and follow-up measurements**.

Parameter	Estimate	Std. error	*p*-Value
Intercept	−3.96	2.90	0.18
Stimulation	1.46	0.74	0.05
Time	0.39	0.10	<0.01
Quadrant	3.27	0.74	<0.01
Sequence	5.99	0.98	<0.01
Time × sequence	−0.18	0.07	0.01
Variance components		SD	
Subjects		6.72	

**Figure 4:**
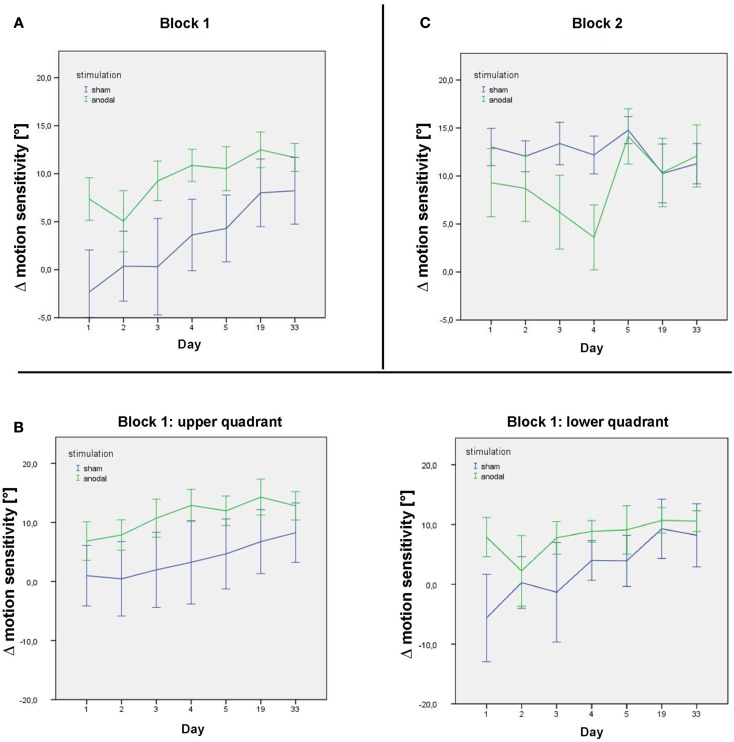
**Δ motion sensitivity during the stimulation week and follow-up**. **(A)** Data from the post-stimulation motion detection tests on days 1–33 in the first study block, showing improvement in age-matched motion detection [subtraction of the subjects’ motion threshold from decade-sensitive normal reference values data = Δ motion sensitivity (in°)] in both the sham (blue) and anodal (green) conditions during the stimulation week and follow-up. **(B)** Data from the post-stimulation motion detection tests on days 1–33 in the first study block for the upper and lower quadrant separately. **(C)** Data from the post-stimulation motion detection tests on days 1–33 in the second study block, showing maintenance of motion detection level in the sham condition (blue) over the intervention week (following anodal stimulation in block 1) and improvement in motion detection in the anodal condition (green) over the intervention week (following sham stimulation in block 1). Follow-up sessions show a similar decrease in motion detection in both groups. Error bars display one standard error of the mean.

The covariate “stimulation” was significant (*p* = 0.05), as were the covariates “time”, “sequence”, and “quadrant” (all *p* < 0.01); importantly, this confirms a significant long-term effect of serial anodal tDCS, again with higher levels of Δ motion sensitivity measured in the upper visual field (Figure 4).

The two-way interaction “sequence × time” yielded a significant interaction (*p* < 0.01), with a negative β-estimate indicating a ceiling effect of learning, declining within the follow-up period after the second block, which was not seen after the first block (Figure 4).

Improved performance did not decline between studies blocks, i.e., subjects started on a higher performance at the beginning of the second stimulation week. Subjects receiving sham tDCS in the second stimulation block, i.e., after the anodal tDCS block, could improve their performance across the second stimulation week to a lesser degree. This might be explained by a ceiling effect of the performance of the motion detection task.

None of the correlation analyses yielded a significant correlation between the stimulation effect (over the stimulation week or the follow-up period) and lesion specific parameters, i.e., the lesion volume or the minimum distance from the internal surface of the occipital pole cranium, and the stimulation effect (*p* > 0.05).

## Discussion

### Summary of results

Serial anodal tDCS over the visual cortex has an additive effect on the performance of a visual perceptual detection task, measuring Δ motion sensitivity in the unaffected hemisphere of subjects with chronic stroke who received tDCS over their lesioned visual cortex. This effect was additional to a fast perceptual learning effect that was particularly present in the early phase of the experiment, but importantly was still measurable in the follow-up measurements 2 and 4 weeks after the last stimulation. Δ motion sensitivity could, however, still be increased by serial anodal tDCS in the later phase of the experiment in the group that received sham tDCS in the first block and in which the learning curve had already reached a plateau. No immediate effects of anodal tDCS on motion sensitivity were seen, and performance improvement was mediated through an overnight effect during the stimulation week.

### Alternative accounts

Alternative explanations for the lack of an immediate stimulation effect might be that Δ motion sensitivity was measured too late. Depending on randomization, Δ motion sensitivity was measured 7–11 min after the end of the application of tDCS. However, this short delay can be considered unlikely to conceal a tDCS effect due to the findings of Antal et al. (2004b), who reported that with a less intense stimulation procedure of 1 mA for 10 min over V1, anodal tDCS led to a reduced moving TMS-phosphene threshold, a measure of excitability of MT, which was still significant 10 min after the anodal tDCS. Pre-existing differences in the subject groups caused by unsatisfactory randomization (e.g., variation in lesion characteristics) are unlikely, since baseline differences were non-significant and were additionally controlled for by the within-subject design. Successful double-blinding was achieved. The effect of serial sham tDCS, however, could not be measured directly since a learning effect was also present in the group that received sham stimulation in the first study block. The improvement of Δ motion sensitivity is most likely mediated by a direct stimulation of the contralesional V1 area through direct anatomical connections to V5/MT. In a previous study of our group (Kraft et al., 2010a), the effect of anodal and cathodal tDCS on contrast sensitivity, a V1 task, was unaffected by the precise site of the stimulating electrode, i.e., over the left or the right visual cortex (O1 or O2). This is most probably due to their close vicinity and the coarse focality of standard tDCS. The greater changes in Δ motion sensitivity measured in the upper visual field quadrant (represented below the calcarine sulcus) compared to the lower visual field quadrant (represented above the calcarine sulcus) also support this hypothesis. Given the electrode montage used, a higher current density could be expected in the infracalcarine V1, whereas the angle between the stimulating and reference electrodes is more tangential compared to the supracalcarine V1. Lesion specific parameters, such as the lesion volume and the minimum distance from the internal surface of the occipital pole cranium did not correlate with the stimulation effect of the contralesional unaffected hemifield.

### Long-term effects of serial tDCS interact with perceptual learning processes

These data represent the first report of long-term effects of anodal tDCS on motion sensitivity, albeit without immediate behavioral effects. A possible explanation for this discrepancy may be that serial anodal tDCS interacts with parallel ongoing learning processes, fostering the stimulation effect over time, with both processes putatively mediated by NMDA-receptor-mediated plasticity (Stagg and Nitsche, 2011). The important interaction of learning and tDCS is highlighted in the small number of studies into serial tDCS that have shown encouraging results in the motor system. Serial anodal tDCS administered over M1 in healthy subjects on five consecutive days during motor task practice sessions significantly enhanced the learning of the complex motor skill task versus sham (Reis et al., 2009). In accordance with our pattern of results, gains were not immediate, but seen selectively between anodal stimulations (i.e., as overnight effects), and persisted throughout a 3-month follow-up phase. It may indeed be the complexity of the sequential visual isometric pinch paradigm used by Reis and colleagues, which required concerted activity of several primary and secondary motor regions, that allowed an elevation of cortical excitability to be translated into lasting behavioral effects, when simpler outcome measures such as specific muscle-group MEP thresholds were not potentiated by anodal tDCS repeated at a 24-h interval (Monte-Silva et al., 2013). More specifically, two 13-min sessions of anodal tDCS administered over the motor-cortex with a 3 or 20 min gap between them lowered MEP thresholds, persistent at 24 h; however, if the gap between the two anodal tDCS sessions was longer, at 3 or 24 h, no persisting raised excitability was seen (Monte-Silva et al., 2013). These findings suggest that the combination of tDCS-modulated excitability with learning is crucial and task-dependent, and emphasize the need to look beyond immediate effects in understanding the potential of tDCS in long-term neuromodulation (Reis et al., 2009; Reis and Fritsch, 2011; Stagg and Nitsche, 2011).

### Temporal dynamics of tDCS effects

It is still a matter of debate whether there is a susceptible time window in which anodal tDCS has its optimal effect. Antal et al. (2004c); Antal and Paulus (2008) reported that the effects of tDCS on V5 are learning-phase dependent: cathodal and anodal tDCS both improved the concomitant learning of a complex visuomotor task in the early phase of learning only, but not later, emphasizing the importance of learning to the effects of tDCS on motion perception.

In contrast, the visuomotor learning paradigm of Reis et al. (2009) revealed a cumulative overnight benefit of anodal tDCS over repeated sessions of anodal stimulation. A possible explanation may be differing difficulties between the tasks applied in both studies.

In the present study, where visuomotor learning processes were of minor importance, perceptual learning processes were assumed to be inherently involved. The learning effect was not detectable immediately after stimulation, but evolved overnight. Serial anodal tDCS over V1 interacted with the process of perceptual learning when administered both in an early learning state (first stimulation week) and in a later over-learned state (second stimulation week). In a psychophysical study of the learning dynamics of the visual submodalities used here, the same motion detection task was practised over 5 days in healthy young subjects (Kraft et al., 2010b). Increasing test difficulty by changing the task to a motion discrimination task effectively flattened the steepness of the learning curve. These findings therefore suggest that task demands influence the time dynamics of perceptual learning processes: it is likely that the difficulty of a task in a repeated tDCS paradigm may contribute substantially to the dynamics of the tDCS effects. Improvements in functional output are likely to come through the integration of tDCS-induced plasticity with task learning, training, and other endogenic plasticity mechanisms.

The present study contrasts on several grounds with the four published studies investigating repeated tDCS in the visual system, all describing one small clinical trial (*n* = 4 per treatment condition) into combined anodal tDCS and visual restoration therapy (VRT), which utilized sparser measurement timepoints at baseline and monthly follow-ups (Halko et al., 2011; Plow et al., 2011, 2012a,b). Although significant improvements compared to sham tDCS with VRT were reported, and fMRI correlates of neuroplastic change were demonstrated in a single case study (Halko et al., 2011), potentially representing encouraging indications of neuroplasticity in the lesioned visual cortex, the studies do not greatly inform our understanding of the short- and medium-term timecourse, or nature of isolated tDCS-specific effects in the visual system.

## Conclusion

The temporal dynamics of long-term (days to weeks) tDCS effects are of general importance to the field of tDCS. Here, we provided evidence for long-term changes of Δ motion sensitivity within the stroke-unaffected hemifield induced by serial anodal tDCS that continue to increase during a 4-week follow-up period. Improving the visual perceptual function of residual intact neurons in the visual system may contribute as a compensational strategy for deficient visual function of stroke-related neuronal loss. Future research efforts should examine the clinical relevance of these findings and focus on the closer temporal and spatial dynamics of serial tDCS with a progression to investigating and further understanding the physiology of clinically applied tDCS within functionally impaired neural systems.

## Conflict of Interest Statement

The authors declare that the research was conducted in the absence of any commercial or financial relationships that could be construed as a potential conflict of interest.
